# Supplementary material for: What factors are important to whom in what context, when adults are prescribed hearing aids for hearing loss? A realist review protocol
Source: BMJ Open. 2022 Jul 14;12(7):e059836. doi: 10.1136/bmjopen-2021-059836 (PMC9295669; doi:10.1136/bmjopen-2021-059836)
Supplement: Supplementary data [file bmjopen-2021-059836supp001.pdf]

Supplementary File

Search #1

CINAHL (1994 – present)

	SEARCHES
**S1**	(MH "Hearing Disorders+")
**S2**	(MH "Deafness+")
**S3**	(MH "Rehabilitation of Hearing Impaired+") OR "persons with hearing impairments"
**S4**	(MH "Presbycusis") OR "presbycusis"
**S5**	(MH "Rehabilitation of Hearing Impaired+") OR (MH "Hearing Disorders+") OR "(hearing loss* OR deaf* OR hearing impair* OR hearing disabilit* OR hearing disorder* OR hearing handicap* OR hearing problem* OR presbycus* OR presbyacus* OR auditory rehabilit*).af."
**S6**	(MH "Hearing Aids+")
**S7**	(MH "Hearing Aids+") OR "(hearing aid* OR listening device* OR sound amplif* OR acoustic amplif* OR hearing device*).af."
**S8**	(MH "Patient Compliance+") OR "treatment adherence and compliance"
**S9**	(MH "Patient Compliance+")
**S10**	"(prescri* OR provi* OR complian* OR cooperat* OR co operat* OR non complian* OR noncomplian* OR non adheren* OR nonadheren* OR accept* OR nonaccept* OR satisfaction OR benefit* OR adapt* OR perception* OR use* OR usage OR adopt* OR uptake* OR reject* OR return* OR success* OR orientat* OR take-up OR utilis* OR non-use)" OR (MH "Prescriptions, Non-Drug")
**S11**	S1 OR S2 OR S3 OR S4 OR S5
**S12**	S6 OR S7
**S13**	S8 OR S9 OR S10
**S14**	S11 AND S12
**S15**	S13 AND S14
**S16**	S15 AND ADULT

Cochrane Library (1996 - present)

	SEARCHES
**1**	Hearing Loss
**2**	Deafness
**3**	Persons With Hearing Impairments
**4**	Presbycusis
**5**	hearing loss* or deaf* or hearing impair* or hearing disabilit* or hearing disorder* or hearing handicap* or hearing problem* or presbycus* or presbyacus* or auditory rehabilit*
**6**	Hearing Aids
**7**	hearing aid* or listening device* or sound amplif* or acoustic amplif* or hearing device*
**8**	treatment adherence and compliance
**9**	Patient Compliance
**10**	prescri* or provi* or complian* or cooperat* or co operat* or non complian* or noncomplian* or non adheren* or nonadheren* or accept* or nonaccept* or satisfaction or benefit* or adapt* or perception* or usage or adopt* or uptake* or reject* or return* or success* or orientat* or take-up or utilis* or non-use
**11**	#1 OR #2 OR #3 OR #4 OR #5
**12**	#6 OR #7
**13**	#8 OR #9 OR #10
**14**	#11 AND #12
**15**	#13 AND #14
**16**	#15 "Adult"

EMBASE (1974 – present)

	SEARCHES
1	hearing loss.mp. or exp hearing impairment/
2	deafness.mp. or exp hearing impairment/
3	exp hearing impaired person/
4	Presbycusis.mp. or exp presbyacusis/
5	(hearing loss* or deaf* or hearing impair* or hearing disabilit* or hearing disorder* or hearing handicap* or hearing problem* or presbycus* or presbyacus* or auditory rehabilit*).af.
6	hearing aids.mp. or exp hearing aid/
7	(hearing aid* or listening device* or sound amplif* or acoustic amplif* or hearing device*).af.
8	(treatment adherence and compliance).mp. [mp=title, abstract, heading word, drug trade name, original title, device manufacturer, drug manufacturer, device trade name, keyword heading word, floating subheading word, candidate term word]
9	exp patient compliance/
10	(prescri* or provi* or complian* or cooperat* or co operat* or non complian* or noncomplian* or non adheren* or nonadheren* or accept* or nonaccept* or satisfaction or benefit* or adapt* or perception* or use* or usage or adopt* or uptake* or reject* or return* or success* or orientat* or take-up or utilis* or non-use).af.
11	1 or 2 or 3 or 4 or 5
12	6 or 7
13	8 or 9 or 10
14	11 and 12
15	13 and 14
16	15 and "Adult" [Subjects]

MEDLINE (1950 – present)

	SEARCHES
**S1**	hearing loss
**S2**	deafness
**S3**	persons with hearing impairments
**S4**	Presbycusis
**S5**	hearing loss* OR deaf* OR hearing impair* OR hearing disabilit* OR hearing disorder* OR hearing handicap* OR hearing problem* OR presbycus* OR presbyacus* OR auditory rehabilit*
**S6**	hearing aids
**S7**	hearing aid* OR listening device* OR sound amplif* OR acoustic amplif* OR hearing device*
**S8**	treatment adherence and compliance
**S9**	Patient Compliance
**S10**	prescri* OR provi* OR complian* OR cooperat* OR co operat* OR non complian* OR noncomplian* OR non adheren* OR nonadheren* OR accept* OR nonaccept* OR satisfaction OR benefit* OR adapt* OR perception* OR use* OR usage OR adopt* OR uptake* OR reject* OR return* OR success* OR orientat* OR take-up OR utilis* OR non-use
**S11**	S1 OR S2 OR S3 OR S4 OR S5
**S12**	S6 OR S7
**S13**	S8 OR S9 OR S10
**S14**	S11 AND S12
**S15**	S13 AND S14
**S16**	S15 "adult".sa suba.

PsycINFO (1967 – present)

	SEARCHES
**1**	exp Hearing Disorders/ or hearing loss.mp.
**2**	exp Deaf/ or deafness.mp.
**3**	Persons With Hearing Impairments.mp.
**4**	exp Hearing Disorders/ or Presbycusis.mp.
**5**	(hearing loss* or deaf* or hearing impair* or hearing disabilit* or hearing disorder* or hearing handicap* or hearing problem* or presbycus* or presbyacus* or auditory rehabilit*).af.
**6**	exp Hearing Aids/
**7**	(hearing aid* or listening device* or sound amplif* or acoustic amplif* or hearing device*).af.
**8**	(treatment adherence and compliance).mp. [mp=title, abstract, heading word, table of contents, key concepts, original title, tests & measures, mesh word]
**9**	exp Treatment Compliance/ or Patient Compliance.mp.
**10**	(prescri* or provi* or complian* or cooperat* or co operat* or non complian* or noncomplian* or non adheren* or nonadheren* or accept* or nonaccept* or satisfaction or benefit* or adapt* or perception* or use* or usage or adopt* or uptake* or reject* or return* or success* or orientat* or take-up or utilis* or non-use).af.
**11**	1 or 2 or 3 or 4 or 5
**12**	6 or 7
**13**	8 or 9 or 10
**14**	11 and 12
**15**	13 and 14
**16**	limit 15 to "300 adulthood <age 18 yrs and older>"

PubMED (1996 – present)

	SEARCHES
1	Hearing Loss
2	Deafness
3	Persons With Hearing Impairments
4	Presbycusis
5	(hearing loss* OR deaf* OR hearing impair* OR hearing disabilit* OR hearing disorder* OR hearing handicap* OR hearing problem* OR presbycus* OR presbyacus* OR auditory rehabilit*).af.
6	hearing aids
7	(hearing aid* OR listening device* OR sound amplif* OR acoustic amplif* OR hearing device*).af.
8	treatment adherence and compliance
9	Patient Compliance
10	(prescri* OR provi* OR complian* OR cooperat* OR co operat* OR non complian* OR noncomplian* OR non adheren* OR nonadheren* OR accept* OR nonaccept* OR satisfaction OR benefit* OR adapt* OR perception* OR use* OR usage OR adopt* OR uptake* OR reject* OR return* OR success* OR orientat* OR take-up OR utilis* OR non-use).af.
11	#1 OR #2 OR #3 OR #4 OR #5
12	#6 OR #7
13	#8 OR #9 OR #10
14	#11 AND #12
15	#13 AND #14
16	#15 AND adult

Web of Science (1998 – present)

	SEARCHES
1	ALL=(hearing loss)
2	ALL=(deafness)
3	ALL=(Persons With Hearing Impairments)
4	ALL=(Presbycusis)
5	ALL=((hearing loss* OR deaf* OR hearing impair* OR hearing disabilit* OR hearing disorder* OR hearing handicap* OR hearing problem* OR presbycus* OR presbyacus* OR auditory rehabilit*).af.)
6	ALL=(hearing aids)
7	ALL=((hearing aid* OR listening device* OR sound amplif* OR acoustic amplif* OR hearing device*).af.)
8	ALL=((treatment adherence and compliance))
9	ALL=(Patient Compliance)
10	ALL=((prescri* OR provi* OR complian* OR cooperat* OR co operat* OR non complian* OR noncomplian* OR non adheren* OR nonadheren* OR accept* OR nonaccept* OR satisfaction OR benefit* OR adapt* OR perception* OR use* OR usage OR adopt* OR uptake* OR reject* OR return* OR success* OR orientat* OR take-up OR utilis* OR non-use).af.)
11	#1 OR #2 OR #3 OR #4 OR #5
12	#6 OR #7
13	#8 OR #9 OR #10
14	#11 AND #12
15	#13 AND #14
16	#15 and Adults
